# The Dual Impact of Smartphone App Usage Diversity on Quality of Life: The Moderating Roles of Age and Digital Literacy

**DOI:** 10.3390/ejihpe15110221

**Published:** 2025-10-27

**Authors:** Chiho Ok

**Affiliations:** College of Business Administration, Kangwon National University, Chuncheon 24341, Republic of Korea; chiho.ok@kangwon.ac.kr; Tel.: +82-33-250-6145

**Keywords:** smartphone app usage diversity, quality of life, age, digital literacy

## Abstract

This study investigates how smartphone app usage diversity (SAUD)—defined as the breadth of applications individuals actively engage with—relates to quality of life, and how these effects are conditioned by age and digital literacy. Drawing on Uses and Gratifications Theory and Cognitive Load Theory, we conceptualize SAUD as having both beneficial and detrimental potential, depending on users’ cognitive and demographic characteristics. Using cross-sectional, self-reported data from the annual nationwide surveys on smartphone overdependence in South Korea, we analyzed a final sample of 20,967 adults (48.4% male, 51.6% female; M_age = 46.0, SD = 13.7; age range 20–69). Results demonstrate that SAUD is positively associated with quality of life among younger and digitally literate users, but negatively associated among older adults and those with lower digital literacy, suggesting the presence of conditional effects. The hypothesized three-way interaction between SAUD, age, and digital literacy was not supported. These findings extend the literature by moving beyond simplistic time-based metrics of smartphone use, offering a more differentiated understanding of mobile technology’s impact on well-being. Practically, the study highlights the need for tailored digital literacy programs and policy interventions that recognize demographic and cognitive diversity in technology engagement. Future research should incorporate longitudinal designs and objective behavioral data to further validate these insights.

## 1. Introduction

The widespread integration of smartphones into everyday life has led to increased scholarly attention on how individuals engage with mobile technology (Montag et al., 2015). Traditionally, this research has focused on aggregate, time-based measures such as screen time or usage frequency to assess the potential benefits or harms of smartphone use (Christensen et al., 2016; Griffioen et al., 2021; Randjelovic et al., 2021; Vezzoli et al., 2023). However, such indicators capture only the depth or intensity of use, overlooking the breadth of engagement across different app domains. Smartphones today serve as multifunctional platforms for communication, productivity, entertainment, and health, among others, making it important to consider not just how much they are used, but how diversely they are used (Falaki et al., 2010; Hartanto et al., 2023; Li et al., 2021).

Although recent studies have begun to emphasize the multidimensional nature of smartphone use, they have not explicitly conceptualized or systematically examined the diversity of app usage as a distinct construct. Prior work has largely treated smartphone use as homogeneous, relying on measures of duration or frequency and thereby overlooking how the breadth of app engagement may differentially influence well-being. To address this limitation, the present study introduces the concept of smartphone app usage diversity (SAUD), defined as the distribution of applications that individuals actively use across various domains of daily life. SAUD offers a more nuanced lens for understanding digital behavior by capturing the breadth of smartphone engagement that complements conventional time-based metrics.

Building on theoretical perspectives, SAUD can be expected to carry both positive and negative implications for quality of life. On the one hand, greater diversity in app use may enhance well-being by allowing individuals to meet a wider range of needs, as suggested by Uses and Gratifications Theory (Joo & Sang, 2013; Moon et al., 2022; Ruggiero, 2000; Whiting & Williams, 2013). On the other hand, excessive diversity may impose burdens when it exceeds users’ capacity to manage technological complexity, as highlighted by Cognitive Load Theory (Baxter et al., 2025; Surbakti et al., 2024; Sweller, 2011). This duality suggests that the effects of SAUD are not inherently beneficial or harmful but rather conditional, depending on users’ cognitive and demographic characteristics.

**Figure 1:**
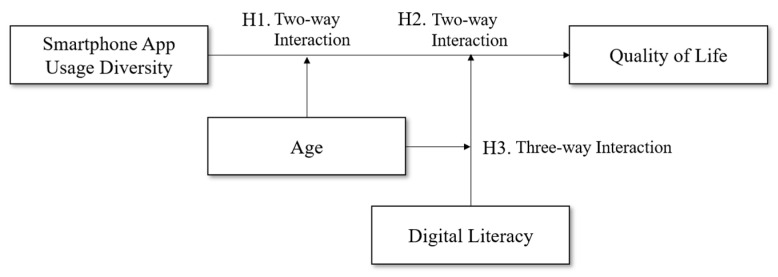
Research model.

To clarify these boundary conditions, the present study investigates the moderating roles of age and digital literacy. Drawing on Socioemotional Selectivity Theory (Bardach et al., 2021; Stevic et al., 2021) and research on technostress (Tarafdar et al., 2011), we argue that these factors shape how individuals cognitively and emotionally respond to diverse app use. Specifically, younger users and those with higher levels of digital literacy are expected to benefit more from SAUD, while older adults and less digitally literate individuals may perceive it as a source of strain or inefficiency (Chen et al., 2025; Oh et al., 2021). By testing both two-way and three-way interaction effects, this study provides a more nuanced understanding of how SAUD contributes to or detracts from quality of life. Beyond its theoretical implications, the findings also suggest the need for tailored digital literacy initiatives that consider demographic and cognitive differences, thereby supporting more adaptive and beneficial technology use. Figure 1 presents the conceptual model proposed in this study.

### 1.1. Smartphone App Usage Diversity

Smartphone app usage diversity (SAUD) refers to the distribution of applications that individuals actively use on their smartphones, moving beyond the conventional focus on total screen time or overall frequency of smartphone engagement. While prior studies have largely emphasized how much time people spend on smartphones as an indicator of potential benefits or risks, this perspective often overlooks the qualitative differences in how smartphones are actually used (Griffioen et al., 2021; Vezzoli et al., 2023). The concept of SAUD highlights the fact that smartphones are multifunctional devices encompassing communication, entertainment, shopping, productivity, and health-related applications. Thus, individuals differ not only in the amount of time they devote to their devices but also in the extent to which they diversify their engagement across various domains (Hartanto et al., 2023; Li et al., 2021). In this sense, SAUD provides a more nuanced understanding of digital behavior by capturing the range of activities embedded in everyday life, reflecting a qualitative dimension that complements traditional time-based measures.

On the one hand, higher levels of SAUD may be associated with positive outcomes for individual user’s quality of life. Drawing from Uses and Gratifications Theory (UGT), individuals actively select media and technological tools to fulfill specific needs, such as acquiring information, maintaining social relationships, or experiencing leisure and entertainment (Joo & Sang, 2013; Moon et al., 2022; Ruggiero, 2000; Whiting & Williams, 2013). By engaging with a diverse set of apps, users are better able to meet these multiple needs, thereby increasing their perceived utility and satisfaction (Hiniker et al., 2016; Ruggiero, 2000; Whiting & Williams, 2013). As a result, individuals with higher SAUD enjoy increased opportunities for learning, networking, and self-development, all of which can positively contribute to overall quality of life. On the other hand, the expansion of app usage diversity may also lead to unintended negative consequences, particularly when it exceeds individuals’ ability to manage technological complexity. From the standpoint of Cognitive Load Theory (Sweller, 2011), human information-processing capacity is inherently limited, and interacting with too many different applications can overwhelm cognitive resources, resulting in confusion, inefficiency, and fatigue (Baxter et al., 2025; Surbakti et al., 2024). In such cases, rather than enhancing utility, diverse app usage can impose additional mental burden and disrupt everyday functioning. This strain may accumulate over time, giving rise to digital fatigue and heightened stress, which ultimately diminishes overall well-being. Accordingly, when smartphone app usage diversity reaches a level that surpasses individuals’ cognitive thresholds, it is reasonable to expect that quality of life will decline rather than improve. Song et al. (2014) highlight the conflicting challenge in mobile application stores of offering a wide variety of apps while ensuring that desired apps are easily discoverable. In a similar vein, SAUD may enhance efficiency and satisfaction by providing broader options, yet it can also induce choice overload and cognitive burden, leading to potential negative outcomes.

Taken together, SAUD is a construct that inherently entails both positive and negative expectations. These perspectives underscore the dual impact of smartphone app usage diversity: while it has the potential to broaden resources and enhance utility or efficiency, it also risks inducing cognitive overload and digital fatigue, which can undermine quality of life. Given this duality, it becomes essential to consider the boundary conditions under which SAUD influences individual outcomes. Prior research across various domains has consistently shown that the effects of diversity—whether in teams (Drach-Zahavy, 2011), network (C. S. Park & Kaye, 2017), or cognition (Kim et al., 2021)—are contingent upon contextual or moderating factors, often producing benefits in some situations while creating drawbacks in others. Building on this insight, the present study assumes that the effect of SAUD on quality of life is not uniform but instead conditional on third factors. Specifically, we focus on age as a key demographic factor and digital literacy as a central cognitive factor, proposing that these characteristics shape whether SAUD contributes to enhanced well-being or leads to diminished quality of life.

### 1.2. Two-Way Interaction: Moderating Role of Age

Socioemotional Selectivity Theory (SST) provides a useful framework for understanding why age plays a critical moderating role in the relationship between smartphone app usage diversity and quality of life. According to SST, individuals’ motivational priorities shift across the life span as perceptions of future time horizons change (Stevic et al., 2021). Younger adults, perceiving time as sufficient, tend to prioritize novelty, information acquisition, and future-oriented goals. In contrast, older adults, who perceive time as more limited, are more motivated by emotionally meaningful goals and a preference for simplicity and stability in daily experiences (Bardach et al., 2021). Within this framework, smartphone app usage diversity may enhance quality of life for younger individuals, as engaging with a broad range of apps supports their pursuit of exploration, learning, and social expansion. However, for older adults, the same diversity may introduce unnecessary complexity, increase cognitive demands, and disrupt their preference for streamlined, emotionally satisfying activities (Busch et al., 2021; Joshi et al., 2020). Thus, SST suggests that while diverse apps may enrich younger individuals’ quality of life, it may conversely undermine quality of life among older individuals by conflicting with their motivational orientation toward simplicity and emotional regulation.

Prior literature suggests that younger individuals are less likely to experience cognitive overload from diverse media use. Nason (2023), for instance, examined the influence of media multitasking, cognitive load, and smartphone addiction on divided attention performance among college students. The experimental study compared heavy and light media multitaskers, as well as individuals meeting the criteria for problematic smartphone use, across tasks with varying levels of cognitive load. The findings indicated no significant cognitive performance differences between groups, nor between control and experimental conditions when an additional video stimulus was presented. These results suggest that younger populations, who typically represent digital natives, may possess a higher tolerance for multitasking and diverse digital engagement, such that increased smartphone app usage diversity does not necessarily impose additional cognitive burden.

While younger individuals may be more resilient to the cognitive demands of diverse app use, older adults are more vulnerable to cognitive overload. Chen et al. (2025) examined the relationship between smartphone use and cognitive function among community-dwelling Chinese older adults aged 60 and above. The study found that while prolonged and diverse smartphone use was generally associated with better overall cognitive function, the type of application used mattered significantly. In particular, social app usage was negatively related to cognitive performance in specific domains, and this effect was especially pronounced among men. These findings suggest that for older adults, engaging with a broader range of applications—particularly socially oriented ones—may increase cognitive demands that exceed processing capacity, thereby impairing cognitive efficiency. Thus, this evidence underscores that smartphone app usage diversity may not always be beneficial for older populations. Instead, it can create additional mental load, reinforcing the expectation that the relationship between app usage diversity and quality of life will be negative among older individuals, in contrast to younger groups who are less susceptible to such cognitive strain. Thus, we hypothesize as follows.

**Hypothesis** **1.** *Age moderates the relationship between smartphone app usage diversity and quality of life such that the relationship would be positive among younger individuals, but the relationship would be negative among older individuals.*

### 1.3. Two-Way Interaction: Moderating Role of Digital Literacy

Digital literacy is widely recognized as a multidimensional construct that extends beyond basic technical skills to encompass the critical, cognitive, and social competencies required for effective engagement in digital environments (Martin & Grudziecki, 2006). Early conceptualizations (e.g., Eshet, 2004) emphasized their multifaceted nature, integrating skills such as photo-visual, reproduction, information, socio-emotional, and real-time thinking literacies. More recent discussions highlight digital literacy as a dynamic and evolutionary concept, shaped by rapid technological advances and intertwined with critical thinking, lifelong learning, and global citizenship (Tinmaz et al., 2022, 2023). Within educational contexts, digital literacy is increasingly viewed as a core component of academic success and professional preparedness, enabling learners to evaluate, create, and share information responsibly (Ng, 2012). At the societal level, digital literacy functions as both an enabler of inclusion and a protective factor against information suppression and disinformation, requiring individuals to navigate digital platforms critically and ethically. In the case of older adults, digital literacy takes on additional significance, as measurement studies indicate that competencies such as content creation and digital safety remain underdeveloped despite growing digital adoption (Oh et al., 2021). Synthesizing these perspectives, digital literacy can be defined as the integrated set of technical, cognitive, and socio-emotional abilities that empower individuals across the lifespan to access, evaluate, create, and communicate information safely and effectively in rapidly evolving digital environments.

Digital literacy is expected to act as a critical boundary condition that determines whether smartphone app usage diversity enhances or undermines quality of life. Individuals with high levels of digital literacy are better equipped to navigate multiple applications, evaluate information effectively, and integrate digital tools into their daily routines. In such cases, diverse app usage can facilitate convenience, efficiency, and access to valuable resources, thereby improving quality of life. Conversely, when digital literacy is low, managing a wide array of applications may generate confusion and stress due to difficulties in evaluating functions, handling technical features, or filtering irrelevant information. The technostress framework suggests that insufficient skills in handling technology amplify strain and reduce perceived benefits, resulting in lower quality of life (Tarafdar et al., 2011). These challenges not only increase cognitive burden but also heighten the likelihood of technostress, ultimately leading to reduced well-being. Thus, digital literacy is likely to moderate the relationship between smartphone app usage diversity and quality of life, such that the relationship is positive when digital literacy is high but negative when digital literacy is low. Thus, we hypothesize as follows.

**Hypothesis** **2.** *Digital literacy moderates the relationship between smartphone app usage diversity and quality of life, such that the relationship is positive when digital literacy is high, but negative when digital literacy is low.*

### 1.4. Three-Way Interaction: Moderating Role of Age and Digital Literacy

Understanding the relationship between SAUD and quality of life requires considering not only individual differences in digital literacy but also age-related factors. We suggest that the impact of SAUD is unlikely to be uniform across the population: individuals with higher digital literacy can leverage multiple applications effectively, whereas those with lower digital skills may experience stress or inefficiency. At the same time, age shapes cognitive resources, motivational priorities, and adaptability to technology, which can further modulate the consequences of diverse app use. By examining a three-way interaction among SAUD, digital literacy, and age, we can capture these sophisticated dynamics, identifying conditions under which app diversity acts as a resource versus a burden. This approach allows for a more precise understanding of how technological engagement translates into well-being across different age groups and skill levels.

When digital literacy is high, SAUD can serve as a resource that enhances quality of life. Individuals with sufficient digital competence are more capable of integrating multiple applications into their daily routines in ways that maximize efficiency, social connectedness, and leisure satisfaction. From a socioemotional selectivity perspective, younger individuals are particularly adept at appropriating diverse apps for goal-directed activities such as information-seeking and entertainment, which directly contribute to their sense of autonomy and well-being. While older adults with high digital literacy can also benefit from diverse app use—particularly through maintaining social contact and managing health-related tasks—the breadth of benefits tends to be greater among younger cohorts who face fewer cognitive constraints and exhibit higher levels of technological adaptability. Thus, the positive association between SAUD and quality of life is expected to be stronger for younger individuals when digital literacy is high.

**Hypothesis** **3a.** *When digital literacy is high, smartphone app usage diversity is positively associated with quality of life for both age groups, and this positive association is stronger among younger than older individuals.*

Conversely, when digital literacy is low, the complexity inherent in managing multiple applications may generate technostress, confusion, and inefficiency, ultimately lowering quality of life. This strain is amplified among older adults, whose reduced cognitive flexibility and greater emphasis on emotionally meaningful goals, as suggested by Socioemotional Selectivity Theory, make it more difficult to adapt to rapid technological demands. In such cases, SAUD is likely to be experienced not as an enabler but as a burden that undermines well-being. Younger individuals with low digital literacy may also encounter negative effects of SAUD, but their generally higher adaptability and more frequent exposure to digital environments buffer some of the strain. Therefore, the negative association between SAUD and quality of life is expected to be stronger for older adults when digital literacy is low.

**Hypothesis** **3b.** *When digital literacy is low, smartphone app usage diversity is negatively associated with quality of life for both age groups, and this negative association is stronger among older than younger individuals.*

## 2. Materials and Methods

### 2.1. Data and Sample

This study drew on data from the nationwide surveys on smartphone overdependence conducted annually in South Korea by the Ministry of Science and ICT in collaboration with the National Information Society Agency (Choi et al., 2017). Initiated in 2004, these surveys have been designed to provide foundational evidence for policy development aimed at fostering responsible smartphone use. The questionnaire items were translated into Korean with reference to relevant academic sources to ensure validity. For the survey, smartphone users were defined as individuals who accessed the internet on their smartphones at least once per month. The sampling frame targeted individuals aged 3 to 69 residing in households across the country as of September of each survey year. A stratified sampling method reflecting regional population distribution was employed, yielding approximately 20,000 respondents from 10,000 households. The 2024 survey adhered to the same methodology and included 24,559 individuals from an equivalent number of households.

From the total of 24,559 respondents, this study focused on adults aged 20 years and older, as minors may differ substantially from adults in terms of quality of life and digital literacy. Cases with missing values on key variables were also excluded. Consequently, the final sample comprised 20,967 individuals. Of this sample, 48.4% were male and 51.6% were female. The mean age was 46 years, ranging from 20 to 69. In terms of occupation, office workers represented the largest group (30.6%), followed by service workers (17.6%), homemakers (12.1%), and sales workers (12.0%), with other categories making up the remainder. Regarding residential area, 41.2% lived in metropolitan cities, 39.8% in mid-sized or small cities, and 19.0% in rural areas. All respondents in the final sample owned a smartphone and reported using it at least once per day.

### 2.2. Variable Measurement

Quality of Life. Quality of Life was measured using four items assessing respondents’ satisfaction with major life domains: interpersonal relationships, work or study, leisure, and overall life (Huang et al., 2025). Participants rated each item on a 4-point Likert scale ranging from 1 (very dissatisfied) to 4 (very satisfied), with higher scores indicating greater perceived quality of life. The four items were: “How satisfied are you with your interpersonal relationships (e.g., family, relatives, friends, neighbors, and colleagues)?” “How satisfied are you with your current work or study?” “How satisfied are you with your leisure activities?” and “Overall, how satisfied are you with your life as a whole?” The reliability of this scale was acceptable (Cronbach’s α = 0.712), supporting the internal consistency of the measure.

Smartphone app usage diversity (SAUD). SAUD was assessed based on self-reported proportions of smartphone use across five application categories—communication, bargaining, leisure, utility and lifestyle, and information and education—as defined by the official questionnaire of the National Information Society Agency’s survey. Respondents were instructed to distribute their daily smartphone use across these categories so that the total equaled 100%. To quantify the degree of diversity in application use, Simpson’s Diversity Index (SDI) was applied, a widely used metric in ecology and social sciences for capturing distributional heterogeneity.

The index is calculated as

$$D=1-\sum_{i=1}^{k}p_{i}^{2}$$

where *p_i_* denotes the proportion of smartphone usage allocated to category *i*, and *k* represents the total number of categories (*k* = 5 in this study). The value of the index ranges from 0 to 1, with higher scores indicating greater diversity in app usage (i.e., more balanced distribution across categories), and lower scores reflecting concentration of use within a limited number of categories.

To demonstrate how the SAUD index reflects the evenness of smartphone app usage, Table 1 presents nine illustrative cases drawn from the sample. Each case shows the proportion of usage across the five categories—Communication, Bargaining, Leisure, Utility and lifestyle, and Information and education—along with the corresponding SAUD value. As shown in Table 1, Case #1, which distributes usage equally across all five categories (20% each), produces the highest SAUD score (0.800). This reflects the fact that the index increases as smartphone use is spread more evenly across categories. By contrast, when usage is more concentrated in one or two categories, the SAUD index declines. For instance, Case #9 allocates 75% of usage to Communication and very little to other categories, resulting in the lowest SAUD score (0.420). Intermediate cases (e.g., Cases from #2 to #8) show how partial imbalances yield moderate diversity scores. These examples clearly illustrate that the SAUD index captures the balance of app usage: higher values indicate more diverse engagement across multiple functions, while lower values signal dependency on a narrower range of applications.

Age. Smartphone users’ age was derived from the self-reported year of birth provided by each respondent. To ensure consistency, participants’ ages were calculated as the difference between the survey year (2024) and the reported birth year. This approach allowed for precise age estimation across all respondents and ensured comparability with previous surveys that employed the same method.

Digital literacy. Digital literacy was assessed using a six-item questionnaire, which represents a shortened version of the scale originally developed by Ng (2012). The measure captured multiple dimensions of digital literacy, including internet searching, evaluating content, communicating information, and creating digital materials. All items were rated on a four-point Likert scale (1 = strongly disagree, 2 = disagree, 3 = agree, 4 = strongly agree). The scale demonstrated good internal consistency, with a Cronbach’s alpha of 0.838.

Controls. Several sociodemographic and behavioral variables were included as controls. Gender was coded as a dummy variable (male = 1, female = 0). Gender was included as a control variable to account for potential unobserved heterogeneity in smartphone usage behavior. Previous studies have reported meaningful gender differences—for example, men tend to use gaming applications more frequently, whereas women are more active in social networking service (SNS) use. Controlling for gender therefore helps to minimize bias stemming from these behavioral differences. Education was measured as the number of years required to reach the respondent’s highest level of education. Family size represented the number of household members living in the same generation. Income referred to total household income, measured in units of 1 million Korean won (e.g., 1 for 1 million KRW, 2 for 2 million KRW, etc.). Problematic smartphone use (PSU) was assessed using the Smartphone Overdependence Scale (S-scale; J. H. Park & Park, 2021), which consists of three subscales: self-control failure, salience, and serious consequences. “Self-control failure” refers to the inability to regulate smartphone use according to personal goals; “salience” captures the extent to which smartphone use dominates daily life; and “serious consequences” measures the negative physical, psychological, and social outcomes associated with excessive use. Ten self-reported items were rated on a four-point Likert scale (1 = strongly disagree to 4 = strongly agree), with higher scores indicating more severe PSU (Cronbach’s α = 0.873). Digital detox was measured by asking respondents whether they had ever deliberately attempted to reduce or control excessive smartphone use, coded as 1 for yes and 0 for no. Regional dummies accounted for place of residence, coded into metropolitan, mid-sized/small city, and rural areas. Housing dummies controlled for the type of residence, including detached houses, apartments, multi-family dwellings and others. Finally, job dummies captured respondents’ occupation across 14 categories, coded as separate dummy variables.

**Construct validity of the multi-item scales.** To assess the construct validity of the multi-item measures used in this study—quality of life, problematic smartphone use, and digital literacy—an exploratory factor analysis (EFA) with varimax rotation was conducted. The analysis extracted three distinct factors corresponding to these constructs, with minimal cross-loadings. All six digital literacy items loaded on a single factor (loadings = 0.69–0.75; eigenvalue = 3.41), accounting for 17.0% of the total variance (see Table 2). The internal consistency coefficient (Cronbach’s α = 0.84) indicated acceptable reliability. These results suggest that digital literacy items form a unidimensional construct and demonstrate adequate construct validity beyond internal consistency.

### 2.3. Procedure and Data Analysis

Since the dependent variable in this study—quality of life—is continuous, ordinary least squares (OLS) regression analysis was used to examine the relationships among the variables. To investigate the moderating roles of age and digital literacy in the relationship between SAUD and quality of life, a hierarchical regression analysis was conducted in four steps. Model 1 included control variables such as gender, age, education, family size, income, problematic smartphone use, digital detox, regional dummies, housing dummies, and job dummies to establish a baseline model. Model 2 added the SAUD variable to assess its direct effect on quality of life. Model 3 added the interaction term between SAUD and age to test Hypothesis 1. Model 4 added the interaction term between SAUD and digital literacy to test Hypothesis 2. Finally, Model 5 incorporated both the two-way interaction terms between SAUD, age, and digital literacy, and the three-way interaction term among three variables to test Hypothesis 3. To reduce multicollinearity, all interaction terms were mean-centered before conducting the regression analysis. All statistical analyses were performed using STATA version 17.0.

## 3. Results

### 3.1. Descriptive and Correlation Analysis

Table 3 presents descriptive statistics and the correlation matrix for the study variables. The mean value of SAUD was 0.732 (SD = 0.077), indicating a moderate level of diversity in smartphone application use among the respondents. Quality of life and digital literacy showed a moderate positive correlation (r = 0.404, *p* < 0.001), suggesting that higher digital competence is associated with greater perceived quality of life. SAUD was positively correlated with digital literacy (r = 0.271, *p* < 0.001) and quality of life (r = 0.078, *p* < 0.001). Importantly, SAUD exhibited low correlations with problematic smartphone use (PSU; r = 0.191, *p* < 0.001) and digital detox (r = 0.123, *p* < 0.001). This pattern indicates that SAUD, as a newly introduced variable in this study, is largely distinct from indicators of smartphone overuse and self-regulation behaviors, supporting its discriminant validity. In other words, the low correlations suggest that app usage diversity captures a unique aspect of smartphone behavior that is not simply a reflection of overdependence or digital self-control efforts. Other control variables behaved as expected: age was negatively associated with digital literacy (r = −0.432, *p* < 0.001) and SAUD (r = −0.186, *p* < 0.001), indicating that younger individuals tend to be more digitally competent and to use a more diverse range of applications.

### 3.2. Hierarchical Regression Analysis

The results of the hierarchical regression analyses are presented in Table 4. Model 1 included only control variables. Model 2 explores the direct effects of SAUD on the quality of life, along with several control variables.

**Figure 2:**
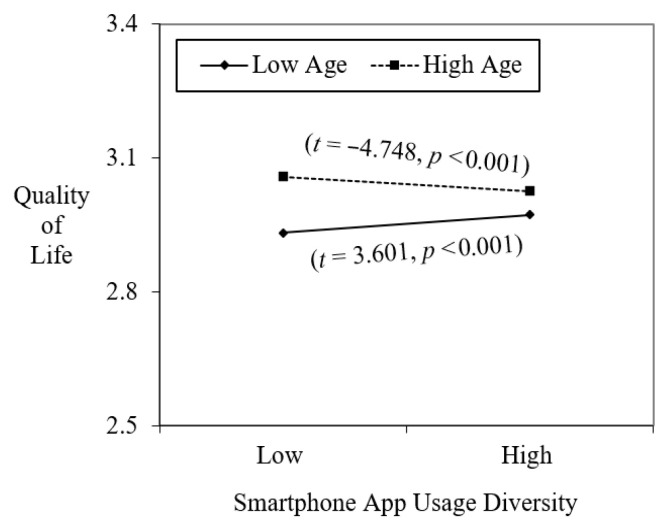
Moderating role of age on the relationship between SAUD and quality of life.

Model 3 introduced the interaction between SAUD and age to test Hypothesis 1, which proposed that age would moderate the relationship between SAUD and quality of life. The results supported this hypothesis, as the interaction term between SAUD and age was significant (b = −0.017, *p* < 0.001). Although the effect size was modest, the negative coefficient indicates a meaningful difference across age groups. Figure 2 presents the interaction pattern, showing that the association between SAUD and quality of life is positive among younger adults (−1 SD from the mean age, approximately 33 years) but slightly negative among older adults (+1 SD, approximately 59 years). In practical terms, this suggests that a one-unit increase in SAUD predicts a small but tangible improvement in younger adults’ perceived quality of life, whereas older adults experience a mild decrease. These findings support Hypothesis 1 and highlight that excessive app diversity may impose cognitive or emotional burdens on older users.

**Figure 3:**
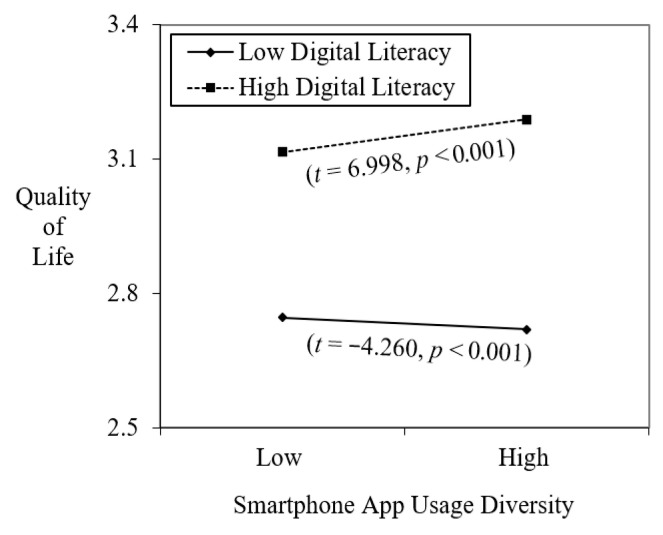
Moderating role of digital literacy on the relationship between SAUD and quality of life.

Model 4 added the interaction between SAUD and digital literacy to test Hypothesis 2. As predicted, digital literacy significantly moderated the relationship between SAUD and quality of life (b = 0.509, *p* < 0.001). This relatively large interaction coefficient indicates a substantial difference in the impact of SAUD depending on individuals’ digital skills. Figure 3 illustrates that for individuals with high digital literacy, greater app usage diversity was strongly and positively associated with quality of life (t = 6.998, *p* < 0.001). In contrast, for those with low digital literacy, the relationship was negative (t = −4.260, *p* < 0.001). This pattern suggests that diverse app engagement enhances well-being only when users possess sufficient digital competence to manage multiple platforms effectively. Thus, the results provide clear support for Hypothesis 2 and demonstrate the protective role of digital literacy in smartphone-based well-being.

**Figure 4:**
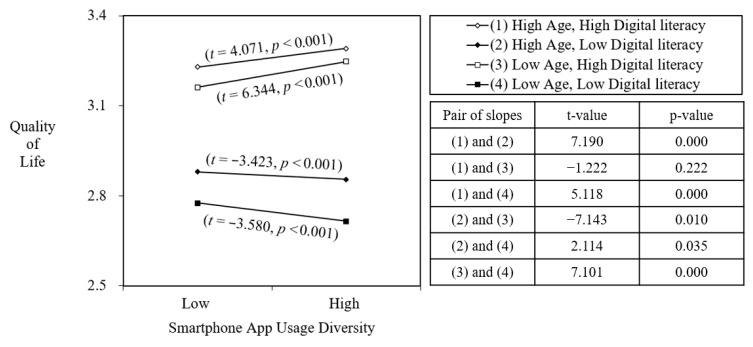
Three-way interaction among SAUD, age and digital literacy on quality of life.

In Model 5, we tested Hypothesis 3 by including the two-way interaction terms as well as the three-way interaction term. Hypothesis 3 proposed that the relationship between SAUD and quality of life would be jointly moderated by age and digital literacy. The results show that the three-way interaction term was statistically significant (b = −0.011, *p* < 0.01). To confirm support for the three-way interaction hypothesis, we plotted the result and conducted slope difference test between each condition. Figure 4 presents the plotted graph based on the analysis results.

We first examined the results for Hypothesis 3a, which predicted that when digital literacy is high, SAUD would be positively associated with quality of life for both age groups, with a stronger positive association among younger than older individuals. In Figure 4, line (1) represents the condition of high digital literacy and high age, while line (3) represents high digital literacy and low age. The result of difference test between the two lines showed that the difference was not statistically significant (t = −1.222, *p* = 0.222). Therefore, Hypothesis 3a was not supported.

Next, we examined Hypothesis 3b, which predicted that when digital literacy is low, SAUD would be negatively associated with quality of life for both age groups, with a stronger negative association among older than younger individuals. In Figure 4, line (2) represents low digital literacy and high age, while line (4) represents low digital literacy and low age. Both lines are negatively associated with quality of life, and the difference test between the two lines revealed a statistically significant difference (t = 2.114, *p* < 0.05). However, contrary to the prediction, when digital literacy was low, the negative effect of SAUD on quality of life was stronger among younger rather than older individuals. Therefore, Hypothesis 3b was not supported.^1^ Although contrary to expectations, this finding suggests that younger individuals with limited digital literacy may be more vulnerable to the cognitive and emotional demands of managing diverse apps, perhaps due to higher multitasking tendencies and heavier exposure to social media. Accordingly, Hypothesis 3b was not supported, but the result provides an interesting nuance for understanding differential vulnerability to digital complexity. These patterns are discussed in detail in the Implications section.

## 4. Discussion

### 4.1. Research Summary

The present study sets out to examine how SAUD relates to quality of life, and whether this relationship is contingent upon age and digital literacy. The findings provide partial support for the proposed hypotheses. First, in line with Cognitive Load Theory and Socioemotional Selectivity Theory, smartphone users’ age emerged as an important moderator: older adults were more vulnerable to the potential cognitive and emotional burden imposed by excessive app diversity, which in turn undermined their quality of life. Second, consistent with the technostress framework, digital literacy played a protective role. Higher levels of digital literacy enabled individuals to leverage diverse app use in ways that enhanced productivity and social connection, thereby improving quality of life, whereas lower literacy exacerbated technostress and reduced the benefits of app diversity. However, contrary to expectations, the hypothesized three-way interaction was not supported. Instead, the results revealed that when digital literacy was low, younger rather than older adults experienced a stronger negative impact of SAUD on quality of life. This finding challenges the assumption that younger adults are uniformly advantaged in navigating digital complexity and suggests that limited digital literacy may represent a hidden vulnerability for younger populations in the smartphone era.

### 4.2. Theoretical Implications

This study aligns with the overarching goal of the Special Issue, which seeks to provide a more optimistic and balanced perspective on the role of technology in shaping mental health and well-being. Traditionally, discussions around digital technology have focused on its potential harms—ranging from issues of addiction, social media-induced stress, to concerns about screen time and cognitive decline (Twenge, 2019; Kuss & Griffiths, 2017). However, this study shifts the focus towards the positive aspects of technology, specifically smartphone apps, and the conditions under which digital engagement may contribute to improving quality of life. By exploring the concept of smartphone app usage diversity and its conditional effects, the study highlights that technology’s impact is far from uniform and is significantly influenced by individual characteristics, such as age and digital literacy. This approach not only enriches our understanding of digital engagement but also invites researchers to reconsider how the intersection of technology, age, and literacy shapes psychological outcomes in today’s digital age. The study emphasizes that with the right conditions, such as higher digital literacy and optimal app diversity, mobile technology can indeed serve as a tool for enhancing well-being (Bardach et al., 2021; Tarafdar et al., 2011). By examining both the positive and negative impacts of technology use, this research contributes to a more holistic narrative that resonates with the aims of the Special Issue.

The theoretical contribution of this study lies in its comprehensive conceptualization of smartphone app usage diversity, which represents a more sophisticated metric for assessing digital engagement than traditional measures such as screen time or app usage frequency (Griffioen et al., 2021; Li et al., 2021). While much of the existing literature focuses on the quantity of smartphone use or the potential for addiction (Kuss & Griffiths, 2017), this study introduces a critical qualitative dimension, emphasizing the variety of apps used across different domains such as communication, entertainment, productivity, and health. This shift in focus allows for a richer understanding of how technology integrates into the daily lives of users and its impact on overall well-being. Theoretically, SAUD connects to Uses and Gratifications Theory (Joo & Sang, 2013; Moon et al., 2022; Raacke & Bonds-Raacke, 2008; Ruggiero, 2000; Whiting & Williams, 2013), which posits that individuals actively select technologies to fulfill their specific needs, whether informational, social, or emotional. By using a diverse range of apps, individuals are better equipped to satisfy these varied needs, which in turn can enhance their perceived quality of life (Hiniker et al., 2016). However, this study also acknowledges the dual nature of SAUD: while it can provide cognitive and emotional benefits, excessive app use diversity may lead to overload, stress, and inefficiency (Baxter et al., 2025; Surbakti et al., 2024). Thus, this conceptualization of SAUD serves to deepen our understanding of digital behavior and offers a framework for exploring how the diversity of mobile technology use impacts psychological outcomes.

This study’s findings contribute to the growing body of literature examining how age influences the cognitive and emotional responses to technology use. Drawing on Socioemotional Selectivity Theory, which posits that individuals’ motivational priorities shift across the life span in response to changing perceptions of time (Carstensen, 1992; Stevic et al., 2021), this study provides empirical evidence that age moderates the relationship between smartphone app usage diversity and quality of life. Specifically, younger adults, with their focus on exploration, novelty, and information seeking, are more likely to benefit from the diversity of apps available on their smartphones. For them, engaging with a variety of applications aligns with their developmental goals, enhancing opportunities for social interaction, learning, and self-expression (Bardach et al., 2021). In contrast, older adults, who prioritize emotionally meaningful goals and seek stability and simplicity in their daily lives, may find the same diversity overwhelming or disruptive (Joshi et al., 2020). This theoretical insight advances the understanding of digital engagement by demonstrating that its impact on well-being is not one-size-fits-all. Instead, it is contingent upon the individual’s age-related goals, motivations, and cognitive capacities. The study thus calls for future research to explore age-specific interventions that can maximize the benefits of mobile technology while minimizing potential negative effects, particularly for older populations.

Another key theoretical implication of this study is the recognition of digital literacy as a critical cognitive resource that moderates the effects of smartphone app usage diversity on quality of life. Digital literacy, which encompasses not only technical skills but also cognitive and socio-emotional competencies (Ng, 2012; Eshet, 2004), plays a crucial role in how individuals engage with diverse mobile applications (Taskin & Ok, 2022). This study demonstrates that individuals with higher digital literacy are better equipped to navigate a broad range of apps, which allows them to integrate technology into their lives in a way that maximizes efficiency, convenience, and overall well-being. On the other hand, those with lower digital literacy may struggle to manage the complexity of multiple apps, leading to confusion, stress, and reduced well-being (Tarafdar et al., 2011). These findings are consistent with the technostress framework, which suggests that insufficient digital competence increases strain and decreases the perceived benefits of technology (Tarafdar et al., 2011). By highlighting the moderating role of digital literacy, this study underscores the importance of digital competence as a resource that not only facilitates technology use but also mitigates potential negative effects, thereby supporting efforts to improve digital literacy education and training across age groups.

While the three-way interaction between SAUD, age, and digital literacy was not statistically significant, the pattern illustrated in Figure 4 showed an intriguing tendency: among the low digital literacy group, younger individuals experienced a more significant decline in their quality of life compared to their older counterparts. This result challenges the assumption that younger populations—often labeled as “digital natives” (Prensky, 2001)—are inherently more adept at handling the cognitive and emotional demands of technology use. This unexpected pattern is consistent with emerging evidence showing that limited digital literacy can negatively influence well-being even among younger or middle-aged adults (Bae, 2022; Sun et al., 2025; Theophilou et al., 2024). These studies collectively suggest that insufficient digital competence may lead to stress, reduced self-efficacy, and lower life satisfaction across age groups. Although younger individuals are generally more familiar with technology, their lower levels of digital literacy may hinder their ability to effectively manage the complexity of diverse app usage. This is particularly concerning given that younger individuals are often at the forefront of digital media consumption and multitasking, which can lead to greater cognitive load (Nason, 2023; Shopova, 2014). Therefore, the study suggests that digital literacy should not be assumed to be an automatic advantage for younger users; instead, even younger populations can experience significant stress and inefficiency if their digital skills are not sufficiently developed. These findings advocate for the inclusion of digital literacy training and interventions for younger generations to help them navigate the complexities of digital environments, ultimately enhancing their quality of life and well-being (Chen et al., 2025; Oh et al., 2021).

Although the findings offer valuable theoretical insights, they should be interpreted with caution given the cross-sectional, self-reported nature of the data. Additionally, as the data were drawn from South Korea—a highly connected and technologically advanced society—the observed relationships may partly reflect culturally specific patterns of digital behavior. Future cross-cultural research is encouraged to test the generalizability of these results.

### 4.3. Practical Implications

From a practical perspective, this study provides valuable insights for developers, educators, and policymakers in designing interventions and strategies that optimize the benefits of smartphone technology for individuals of different ages and digital literacy levels. For technology developers, the findings suggest that offering diverse, user-friendly apps that cater to a variety of needs can enhance user satisfaction and well-being, especially for younger individuals and those with higher digital literacy. However, developers should also consider features that simplify app usage for older adults and those with lower digital literacy to prevent cognitive overload and technostress. Educational institutions and training programs can utilize these findings to design digital literacy curricula that are age-appropriate and responsive to the evolving technological landscape (Lee, 2014). Given the critical role that digital literacy plays in moderating the effects of smartphone app usage diversity, targeted educational programs can help individuals navigate digital spaces more efficiently, fostering positive outcomes for mental health and well-being. From a policy standpoint, governments and public health agencies should prioritize digital literacy as part of broader strategies to reduce the digital divide and improve social inclusion, particularly for older adults and vulnerable populations. Implementing national campaigns that promote digital literacy across the lifespan could empower individuals to manage technology more effectively, thus enhancing their overall quality of life. Moreover, policymakers should consider supporting the development of age-specific apps or digital interventions that are tailored to the cognitive and emotional needs of different demographic groups. These initiatives would help ensure that technology continues to serve as a tool for well-being rather than a source of stress or disengagement.

### 4.4. Limitations and Future Research

Although this study provides valuable insights, it is subject to several limitations that should be addressed in future research. First, the reliance on self-reported survey data presents potential biases, particularly in the estimation of smartphone usage distribution. Participants may have underreported behaviors that they perceive as excessive or detrimental due to social desirability bias. Future studies should aim to incorporate more objective data sources, such as digital trace data or app usage logs, to enhance the accuracy and reliability of the findings. Second, while this study used nationwide cross-sectional data, it did not employ a panel design. As a result, repeated measures from the same individuals over time were not available, which limits the ability to examine intra-individual changes. A longitudinal study or lagged design would be more appropriate for tracking the evolution of smartphone usage patterns and for drawing more robust conclusions about causal relationships. Specifically, collecting independent and dependent variables at different time points would improve the temporal order necessary for causal inference. Third, this study focused on only five categories of smartphone apps—communication, bargaining, leisure, utility and lifestyle, and information and education. However, the rise of other app categories, such as video streaming and short-form content platforms, calls for a broader examination of app usage. Future research should incorporate a wider variety of app types to offer a more comprehensive understanding of the impacts of app-based engagement on problematic smartphone use. Sixth, despite the inclusion of multiple sociodemographic and behavioral controls, a considerable proportion of variance in quality of life remained unexplained. This outcome is expected in large-scale social science studies, as quality of life is influenced by numerous factors not captured in our dataset, such as employment status, family responsibilities, and health conditions. Given that the survey focused on smartphone-related behaviors, these broader determinants were not measured, and the heterogeneity of our large national sample further contributed to unexplained variance. Future research should incorporate additional psychosocial and health-related variables and focus on specific subgroups to achieve greater explanatory power. Lastly, the generalizability of these findings is limited by the exclusive focus on South Korea, one of the world’s most digitally connected countries with high smartphone penetration rates. Therefore, the results may not be directly applicable to other cultural or technological contexts. Comparative studies in different countries are needed to test the cross-cultural validity of the observed relationships.

## 5. Conclusions

In conclusion, this study underscores the importance of individual characteristics such as age and digital literacy in moderating the relationship between smartphone app usage diversity and quality of life. The results demonstrated that the two-way interactions between SAUD and age, and between SAUD and digital literacy, were both significant, supporting the proposed moderating roles of these variables. However, the hypothesized three-way interaction among SAUD, age, and digital literacy was not supported, indicating that the combined effect of these factors did not significantly explain additional variance in quality of life. Younger individuals and those with higher digital literacy are more likely to experience positive outcomes from diverse app usage, while older adults and those with lower digital literacy may face cognitive overload and stress. By examining both two-way and three-way interactions, the study provides a deeper understanding of how SAUD affects well-being across different demographic groups. These findings highlight the need for targeted digital interventions and policies to improve smartphone use and digital literacy, particularly for vulnerable populations. Future research should address the study’s limitations, including incorporating objective data sources and broader app categories, to further refine our understanding of technology’s impact on quality of life.

## Figures and Tables

**Table 1:** Examples of Smartphone app usage diversity measure.

Smartphone App Categories and Relative Usage Ratio	Case Examples (Selective)								
	#1	#2	#3	#4	#5	#6	#7	#8	#9
Communication	20	20	25	30	46	60	20	50	75
Bargaining	20	15	0	20	9	10	5	0	10
Leisure	20	30	25	40	27	10	65	0	5
Utility and lifestyle	20	20	25	5	0	10	5	0	5
Information and education	20	15	25	5	18	10	5	50	5
SAUD index	0.800	0.785	0.750	0.705	0.675	0.600	0.530	0.500	0.420

**Table 2:** Validity test using exploratory factor analysis.

Variables	Items	Factor 1	Factor 2	Factor 3	Reliability
Quality of life	Item_01	−0.023	0.242	0.612	0.712
	Item_02	−0.021	0.229	0.656	
	Item_03	−0.019	0.114	0.717	
	Item_04	−0.034	0.171	0.792	
Problematic smartphone use	Item_01	0.710	0.129	−0.095	0.881
	Item_02	0.683	0.137	−0.152	
	Item_03	0.670	0.172	−0.168	
	Item_04	0.687	0.159	−0.106	
	Item_05	0.696	0.128	−0.032	
	Item_06	0.688	0.124	−0.006	
	Item_07	0.681	0.038	0.059	
	Item_08	0.686	−0.011	0.112	
	Item_09	0.693	−0.023	0.127	
	Item_10	0.720	−0.056	0.067	
Digital literacy	Item_01	0.056	0.707	0.168	0.838
	Item_02	0.038	0.720	0.145	
	Item_03	0.081	0.754	0.151	
	Item_04	0.138	0.704	0.206	
	Item_05	0.098	0.754	0.128	
	Item_06	0.146	0.688	0.097	
Eigenvalue		4.845	3.405	2.194	
% of Variance		24.226	17.024	10.970	
% Cumulative		24.226	41.249	52.220	

**Table 3:** Descriptive statistics and correlation matrix.

Variables	1	2	3	4	5	6	7	8	9	10
1. Quality of life	(0.712)									
2. SAUD	0.078 **	1.000								
3. Digital literacy	0.404 **	0.271 **	(0.838)							
4. Age	−0.113 **	−0.186 **	−0.432 **	1.000						
5. Gender	0.020 *	0.010	0.068 **	0.024 **	1.000					
6. Education	0.168 **	0.207 **	0.438 **	−0.618 **	0.106 **	1.000				
7. Family size	0.064 **	0.097 **	0.181 **	−0.300 **	0.014	0.252 **	1.000			
8. Income	0.118 **	0.130 **	0.245 **	−0.215 **	0.026 **	0.265 **	0.533 **	1.000		
9. PSU	−0.028 **	0.191 **	0.232 **	−0.247 **	0.030 **	0.199 **	−0.077 **	0.101 **	(0.881)	
10. Digital detox	0.123 **	0.123 **	0.227 **	−0.106 **	−0.011	0.105 **	0.049 **	0.111 **	0.148 **	1.000
Mean	3.008	0.732	2.738	46.003	0.484	14.268	3.019	3.246	1.905	0.524
S.D.	0.481	0.077	0.637	13.713	0.499	2.281	1.077	1.058	0.509	0.499

Note 1. SAUD = Smartphone app usage diversity; PSU = problematic smartphone use. Note 2. The number in parentheses means the Cronbach alpha value. N = 20,967. * *p* < 0.01, ** *p* < 0.001.

**Table 4:** The results of hierarchical regression analyses predicting quality of life.

Variables	Model 1		Model 2		Model 3		Model 4		Model 5	
	b	S.E.	b	S.E.	b	S.E.	b	S.E.	b	S.E.
Constant	2.110 **	0.052	2.171 **	0.058	2.055 **	0.060	1.950 **	0.061	1.955 **	0.064
Regional dummies										
Metropolitan (ref.)										
Mid-sized/small city	−0.011	0.006	−0.010	0.006	−0.010	0.006	−0.011	0.006	−0.007	0.006
Rural areas	0.030 **	0.008	0.030 **	0.008	0.031 **	0.008	0.030 **	0.008	0.035 **	0.008
Housing dummies										
Detached house (ref.)										
Apartments	0.049 **	0.007	0.048 **	0.007	0.049 **	0.007	0.050 **	0.007	0.050 **	0.007
Multi-family dwellings	0.034 **	0.009	0.033 **	0.009	0.034 **	0.009	0.035 **	0.009	0.035 **	0.009
Others	−0.0005	0.021	−0.0007	0.021	−0.0003	0.021	−0.002	0.021	−0.002	0.021
Job dummies										
Managers (ref.)										
Professionals	−0.028	0.034	−0.028	0.034	−0.030	0.034	−0.026	0.034	−0.027	0.034
Clerical workers	−0.038	0.030	−0.039	0.030	−0.040	0.030	−0.039	0.030	−0.042	0.030
Service	−0.076	0.031	−0.076	0.031	−0.074	0.031	−0.072	0.031	−0.070	0.031
Sales	−0.086 *	0.031	−0.086 *	0.031	−0.085 *	0.031	−0.080 *	0.031	−0.079	0.031
Agricultural	−0.037	0.038	−0.039	0.038	−0.040	0.038	−0.034	0.038	−0.039	0.038
Trade	−0.028	0.032	−0.028	0.032	−0.026	0.032	−0.021	0.032	−0.021	0.032
Operate and Assemble	−0.096 *	0.035	−0.096 *	0.035	−0.093 *	0.035	−0.089 *	0.035	−0.090 *	0.035
Unskilled	−0.149 **	0.034	−0.148 **	0.034	−0.148 **	0.034	−0.143 **	0.034	−0.151 **	0.034
Military	−0.191	0.433	−0.190	0.433	−0.189	0.433	−0.186	0.432	−0.187	0.432
Homemakers	−0.072	0.032	−0.073	0.032	−0.073	0.032	−0.072	0.032	−0.073	0.032
Students	−0.014	0.034	−0.016	0.034	−0.017	0.034	−0.015	0.034	−0.028	0.034
Unemployed	−0.106 *	0.037	−0.109 *	0.037	−0.114 *	0.037	−0.118 *	0.037	−0.127 **	0.037
Others	−0.117	0.109	−0.118	0.109	−0.124	0.109	−0.114	0.109	−0.113	0.108
Age	0.003 **	0.0003	0.003 **	0.0003	0.003 **	0.0003	0.003 **	0.0003	0.003 **	0.0003
Gender	−0.021 *	0.006	−0.022 **	0.006	−0.021 *	0.006	−0.022 **	0.006	−0.022 **	0.006
Education	0.005 *	0.001	0.005 *	0.001	0.006 **	0.001	0.006 **	0.001	0.007 **	0.001
Family size	−0.004	0.003	−0.004	0.003	−0.003	0.003	−0.004	0.003	−0.003	0.003
Income	0.012 **	0.003	0.012 **	0.003	0.012 **	0.003	0.012 **	0.003	0.013 **	0.003
PSU	−0.120 **	0.006	−0.118 **	0.006	−0.118 **	0.006	−0.116 **	0.006	−0.116 **	0.006
Digital detox	0.041 **	0.006	0.042 **	0.006	0.041 **	0.006	0.036 **	0.006	0.037 **	0.006
Digital literacy	0.325 **	0.005	0.327 **	0.005	0.330 **	0.005	0.331 **	0.005	0.333 **	0.005
SAUD			−0.101	0.041	0.022	0.045	0.145 *	0.046	0.099	0.051
SAUD × Age					−0.017 **	0.002			0.001	0.003
SAUD × Digital literacy							0.509 **	0.047	0.594 **	0.068
Age × Digital literacy									−0.001 **	0.0003
SAUD × Age × Digital literacy									−0.011 *	0.003
F-value	192.47 **		185.61 **		180.62 **		184.08 **		167.58 **	
R-squared	0.1929		0.1931		0.1945		0.1975		0.1988	
ΔR-squared	0.1929		0.0002		0.0014		0.0030		0.0013	
Adj. R-squared	0.1919		0.1921		0.1935		0.1965		0.1976	

Note. SAUD = Smartphone app usage diversity; PSU = problematic smartphone use. N = 20,967. * *p* < 0.01, ** *p* < 0.001.

## Data Availability

The data of this study is available to anyone under the permission of the National Information Society Agency in South Korea (https://www.nia.or.kr, accessed on 20 October 2025).

## Note

1 Additional analyses were conducted separately for male and female respondents. The results showed the same pattern as those of the overall sample—Hypotheses 1 and 2 were supported, while Hypothesis 3 remained non-significant in both subsamples—indicating that the main findings are consistent across gender.
